# Utility of Bronchoscopy With Endobronchial Ultrasound in Diagnosis and Monitoring of Pulmonary Embolism

**DOI:** 10.7759/cureus.17965

**Published:** 2021-09-14

**Authors:** Joni Chow, Jessica Roberts, Kovid Trivedi

**Affiliations:** 1 Pulmonary Medicine, Western University of Health Sciences, Lebanon, USA; 2 Pulmonary/Critical Care Medicine, Salem Pulmonary Associates/Salem Health, Salem, USA

**Keywords:** bronchoscopy, endobronchial ultrasound (ebus), pulmonary embolism (pe), venous thromboembolism (vte), ct angiography of chest, cardiac arrest

## Abstract

Pulmonary embolism (PE) is a common diagnosis made in the emergency department (ED). Although CT angiogram remains the preferred diagnostic modality, sometimes it is not possible to obtain due to hemodynamic instability, renal failure, pregnancy, etc. Bronchoscopy with endobronchial ultrasound (EBUS) has been used for the evaluation of mediastinal and hilar lesions. The proximity of pulmonary vasculature to mediastinal and hilar structures can aid in utilizing EBUS for bedside diagnosis and future monitoring of pulmonary embolism. This case describes a patient who presented with cardiac arrest, likely secondary to a massive pulmonary embolism, and later underwent bronchoscopy with EBUS that demonstrated the pulmonary emboli.

## Introduction

Diagnosing pulmonary embolism (PE) in a timely and efficient manner is imperative to good patient care and outcomes. Patients presenting with PE typically have nonspecific signs and symptoms broadly ranging from shortness of breath to hemodynamic instability. Traditionally, chest computed tomography angiogram (CTA) is the preferred imaging modality due to its speed, reliability, and ease of access in even the most remote of hospitals. While ventilation-perfusion scans are another diagnostic tool, it is less commonly utilized due to the added time needed to perform the procedure, and for many patients, time is of the essence. Furthermore, patient populations that cannot undergo CTA (clinical instability, pregnancy, or renal failure) are at a distinct risk of delayed diagnosis [1]. Anatomically, the tracheal and bronchial walls lay in proximity to the pulmonary arteries which conveniently allows for bronchoscopy with endobronchial ultrasound (EBUS) to aid in the visualization of clots or other vascular abnormalities [2]. In the past, EBUS has been a primary tool for evaluating mediastinal and hilar lesions [3], but we believe that the capabilities of EBUS outreach its most well-known utilization. Traditionally, EBUS has not been used for PE evaluation due to factors like operator availability in the emergency department (ED), more time requirement than CTA, ability to accurately comment only on larger pulmonary vessels, and inter-operator differences. In this case, we describe findings of a pulmonary embolus on EBUS and propose the use of EBUS as an alternative diagnostic as well as a monitoring tool for emboli resolution in the management of pulmonary embolism. 

## Case presentation

A 47-year-old male presented to the emergency room after a witnessed cardiac arrest at home. The patient and his wife had returned home after vacation, and the patient was mowing his lawn after arrival. During this, the patient started experiencing shortness of breath and went inside the house. The patient suddenly became unresponsive and his wife, being a nurse, identified it quickly and started cardiopulmonary resuscitation (CPR). Upon the arrival of emergency medical services, the patient had a return of spontaneous circulation (ROSC) and was found to be in supraventricular tachycardia. The patient received 6 mg adenosine IV and converted to sinus rhythm. The patient was then alert, awake, and hemodynamically stable, and was brought to the ED for further evaluation. The patient had a history of hypertension and moderate aortic insufficiency. He was a non-smoker with no history of alcohol or illicit drug intake. Surgical history was significant for Achilles tendon repair four months ago.

In the ED, the patient was found to have saddle pulmonary embolism extending to the segmental level pulmonary arteries on CTA. A transthoracic echocardiogram showed right heart strain with normal left ventricular ejection fraction. Troponin was elevated at 0.03 ng/mL. Laboratory analysis was also significant for blood glucose 209 mg/dL, serum creatinine 1.34 mg/dL, serum bicarbonate 16 mmol/L, ALT 99 units/L, AST 105 units/L, total WBC count 11,600 with lymphocytosis. Urine drug analysis was unremarkable. Hemoglobin A1c was 5.4%. COVID-19 PCR was negative. CTA also revealed a 2 cm right upper lobe nodule along with right hilar and paratracheal adenopathy.

With hemodynamic stability, catheter-directed thrombolysis was chosen over systemic thrombolysis. The patient was then admitted to the intensive care unit (ICU) for further management. Over the course of the hospital stay, the patient continued to improve clinically and a decision was made to further evaluate the lung nodule and lymphadenopathy, as it would be more convenient to hold heparin for the procedure compared to an oral anticoagulant as an outpatient.

**Figure 1:**
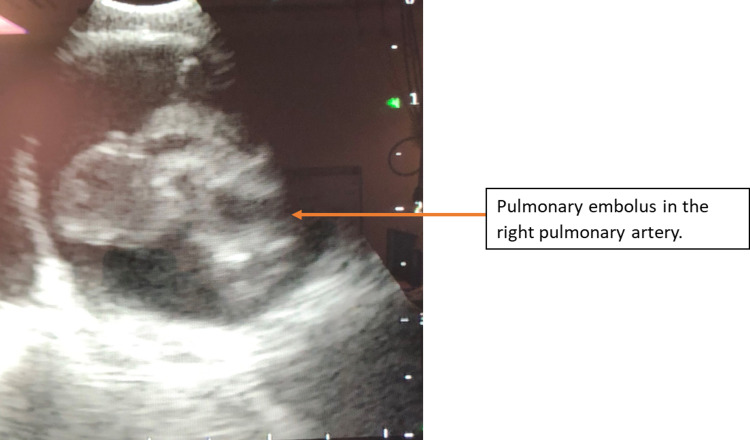
Pulmonary embolus in the right pulmonary artery.

The patient underwent flexible bronchoscopy with endobronchial ultrasound for evaluation of the mediastinal and hilar lymph nodes. During the procedure, a significant clot burden with a mobile clot was seen in the pulmonary artery and its branches by endobronchial ultrasound (Figure 1). The clot had a varied degree of opacification within itself.

## Discussion

Currently, EBUS is primarily used for transbronchial needle aspiration (TBNA) to diagnose mediastinal and hilar lesions, and in lymph node staging of lung cancer, but we propose that EBUS has unexplored potential as a diagnostic tool for identification and management of pulmonary embolism. EBUS has proven itself to be an effective alternative to PE diagnosis in patients who are unable to undergo CTA (hemodynamically unstable, pregnant, chronic renal insufficiency, etc.). It is a modality that can be mobilized quickly to be available at the bedside in ED or ICU. This ultimately broadens the patient population that can timely obtain PE treatment and management. One case study reported that a pulmonary embolism that was missed on CTA was visualized with EBUS, further supporting the untapped potential of EBUS [4]. EBUS is a safe, dependable, and feasible method of diagnosing PE [5], with a 96% sensitivity in detecting PE [6], there are few studies that explore the use of EBUS as a follow-up tool in PE management. Follow-up imaging for PE is not recommended in an acute setting unless the patient remains hemodynamically unstable post thrombolysis, where it might be useful. More commonly, these patients are too unstable to be transported for CTA or have concurrent renal failure due to hemodynamic instability, where IV contrast administration might be detrimental. In these cases, a bedside tool such as bronchoscopy with EBUS might be useful. As our clinical case demonstrates, EBUS was demonstrated to be beneficial not only as a means to visualize the patient’s PE but as a way to monitor the resolution status of the emboli, further influencing patient care and management.

## Conclusions

While EBUS will likely never replace the speed and reliability of CTA, it is another alternative that should be considered in a patient’s diagnostic workup and monitoring, particularly in patients who cannot undergo CTA, and more physicians should be encouraged to complete the necessary procedural training. Future research should focus on exploring the utility of EBUS in the overall diagnosis and management of this relatively common but potentially fatal disease entity. Although it is a bedside tool, it can only recognize large pulmonary emboli while missing subsegmental emboli, which needs to be paid attention to while interpreting results.
